# Race/ethnicity on firearm & self-injuries during COVID-19 using TQIP data

**DOI:** 10.1016/j.sopen.2026.01.001

**Published:** 2026-01-10

**Authors:** Veronica Layrisse Landaeta, Shahenda Khedr, Victoria Yuan, Eshani Pareek, Debra D'Angelo, Elizabeth Zhao, Gala Cygiel, Konstantin Khariton, Steven Y. Chao

**Affiliations:** a Department of Surgery, New York-Presbyterian/Queens, 56-45 Main St, Flushing, NY 11355, USA; b Department of Population Health Sciences, Weill Cornell Medicine, 575 Lexington Avenue, New York, NY, 10022, USA; c Department of Surgery, Weill Cornell Medicine, New York, NY, USA

**Keywords:** COVID-19 pandemic, Assaults, Self-inflicted injuries, Firearm-related injuries, Racial and ethnic disparities, Trauma and critical care

## Abstract

**Introduction:**

The COVID-19 pandemic led to increased assaults, self-inflicted injuries, and firearm-related injuries across the nation, along with increased reports of depression and anxiety worldwide. Our study examines trends in these types of injuries among diverse racial and ethnic groups in the United States during this time.

**Methods:**

Data was extracted from the ACS-TQIP database, including patients with assaults, self-inflicted injuries and firearm-related injuries from 2018 to 2021. Pre-COVID period was defined as 2018–2019, and COVID period as 2020–2021. We performed univariable and multivariable logistic regressions to identify associations between injury rates and COVID period, sex, race, and ethnicity.

**Results:**

We identified 417,797 assaults (9.3% of traumas), 57,853 self-inflicted injuries (1.3%) and 208,882 firearm-related injuries (4.7%). Multivariable analysis revealed significant increase in assaults (OR: 1.02, 95% CI: [1.01, 1.03]; *p* < 0.001) and firearm-related injuries (1.28 [1.27, 1.29]; p < 0.001) and a significant decrease in self-inflicted injuries (0.98 [0.97, 0.99]; *p* = 0.039) during the COVID period.

Asian American Pacific Islanders (1.06 [1.02–1.10]; *p* < 0.005), American Indians (3.47 [3.35–3.60]; *p* < 0.001), Black or African American (5.32 [5.26–5.38]; *p* < 0.001, other races (1.23 [1.20–1.25]; p < 0.001) and Hispanics (1.71[1.65–1.74]; p < 0.001) had higher odds of assaults during COVID.

Asian American Pacific Islanders (1.12 [1.04–1.20]; *p* = 0.001) and American Indians (1.23 [1.12–1.35]; *p* < 0.001 had higher odds of self-inflicted injuries and Hispanic patients had lower odds (0.73 [0.70–0.76]; *p* < 0.001) during COVID.

American Indians (1.45 [1.36–1.55]; *p* < 0.001), Black or African Americans (6.42 [6.33–6.52]; *p* < 0.001), Hispanics (1.46 [1.43–1.50]; p < 0.001) and other races (1.11 [1.08–1.15]; p < 0.001) had higher odds of firearm related injuries during COVID.

**Conclusion:**

The COVID period saw higher odds of assaults, self-inflicted injuries, and firearm-related injuries in certain racial/ethnic minorities. These findings highlight the need for targeted interventions to address the disproportionate impact on racial/ethnic minorities.

## Highlights

• The COVID-19 pandemic was associated with increased assaults and firearm injuries.
• Self-inflicted injury rates showed a modest decline during the COVID period.
• Black and American Indian patients experienced the greatest injury disparities.
• Asian American Pacific Islanders demonstrated elevated self-inflicted injury risk.
• Findings underscore the need for targeted, equity-focused trauma interventions.

## Introduction

The coronavirus disease 2019 (COVID-19) pandemic was a global health crisis of unprecedented scale that reshaped lives and altered the landscape of public health concerns worldwide. In the United States (US), the pandemic's impact was multifaceted, affecting physical health, personal safety, and mental well-being.

The nation witnessed an alarming increase in assaults leading to hospitalizations, signaling a rise in violence amid the pandemic's various stressors [1]. Data from the Council on Criminal Justice highlights that while property crimes declined during the pandemic, other crimes such as robberies, nonresidential burglaries, and homicides rose in smaller cities [[2], [3], [4]]. As society began to reopen and stay-at-home orders were lifted, there was a marked escalation in firearm violence, injuries, and deaths, emphasizing the need for comprehensive public health interventions [5]. Six trauma centers reported increased admissions for gunshot wounds among patients with histories of mental illness and substance use disorders [6]. Illinois hospitals similarly noted a rise in firearm injuries, especially among African Americans, individuals aged 15–34, and Chicago residents, likely due to pandemic-induced social and economic stressors [7].

The pandemic also saw an increase in the frequency and severity of self-harm incidents. Ethanol poisoning was the most common mechanism of self-injury, followed by hanging, defenestration, sharp object injuries, and drowning [8,9]. Notably, self-harm attempts were more present among women aged 10–20 and 41–50, with a history of psychiatric illness and on multiple psychiatric medications [10]. Consequently, emergency department visits for self-harm surpassed pre-pandemic levels, becoming the third most common cause of injury [6,8,11].

Racial and ethnic disparities became even more pronounced, with Blacks, Asian American Pacific Islanders (AAPI), and Hispanics experiencing worsening mental health outcomes compared to Caucasians during the COVID-19 pandemic [7,[12], [13], [14]]. Addressing the rise in assaults, firearm injuries, and declining mental health calls for continued research to examine sociodemographic factors that influence self-harm and gun violence, particularly within racial and ethnic minorities. To the best of our knowledge, this is the first study using the American College of Surgeons' Trauma Quality Improvement Program database to examine the relationship between COVID-19 and trauma among racial/ethnic groups.

## Materials and methods

This is a retrospective cohort study using data extracted from the American College of Surgeons Trauma Quality Improvement Program (TQIP) database. This database is maintained by the American College of Surgeons Committee on Trauma and aggregates de-identified patient data from over 900 trauma centers across the United States. The data contained in the TQIP database are standardized at the time of submission using the validation system and rules defined in the National Trauma Data Standard (NTDS) data dictionary [15]. The study was granted exemption by the Institutional Review Board at New York Presbyterian/Queens.

In the NTDS, assaults and injuries are classified using specific International Classification of Diseases 10th Revision (ICD-10) codes. We identified patients who suffered from assaults, self-inflicted injuries, and/or injuries from firearms between January 1, 2018 and December 31, 2021. Two distinct cohorts were established in relation to the onset of the COVID-19 pandemic: the pre-COVID cohort (years 2018–2019) and the COVID cohort (years 2020–2021). Firearm injuries included those that were self-inflicted and those resulting from assault. Self-inflicted injuries encompassed various mechanisms, including cut/pierce, poisoning, burns, suffocation, falls and drowning/submersion.

Descriptive statistics were employed to summarize the demographic characteristics and outcomes of the overall sample and each cohort. We used univariable logistic regression to examine associations between occurrence of assault, firearm and self-inflicted injuries during the pre-COVID and COVID periods. Variables were then selected for multivariable logistic regression models based on their clinical relevance and statistical significance from the univariable analyses. All statistical analyses were performed using R (version 4.1.1).

## Results

During the study period, a total of 4,483,076 injuries were reported, of which 9.3% were assaults (*n* = 417,797), 1.3% self-inflicted injuries (*n* = 57,853), and 4.7% firearm-related injuries (*n* = 208,882) (Table 1). 9.0% of which were assaults (*n* = 193,588), 1.3% self-inflicted injuries (*n* = 28,026), and 4.0% firearm-related injuries (*n* = 86,538). During the COVID period, 2342.150 trauma injuries were reported. Assaults rose to 9.6% *n* = 224,209) and firearm-related injuries increased to 5.2% (*n* = 122,344). The proportion of self-inflicted injuries during the COVID period remained similar compared to the pre-COVID period at 1.3% (*n* = 29,827) (Table 1).

**Table 1:** Cohort characteristics.

Characteristic	Full cohort	Cohort by time period	
		Pre-COVID (2018–2019)	COVID (2020−2021)
Overall injuries	4,483,076	2,140,962	2342,150
Injury subtype			
Assaults	417,797 (9.3%)	193,588 (9.0%)	224,209 (9.6%)
Self-inflicted	57,853 (1.3%)	28,026 (1.3%)	29,827 (1.3%)
Firearm	208,882 (4.7%)	86,538 (4.0%)	122,344 (5.2%)
Race			
AAPI	98,131 (2.2%)	46,228 (2.2%)	51,903 (2.3%)
American Indian	39,315 (1%)	18,575 (1%)	20,740 (1%)
Black	653,004 (15%)	300,067 (14%)	352,937 (15%)
White	3,276,540 (74%)	1,581,370 (75%)	1,695,170 (74%)
Other	335,422 (7.8%)	159,458 (7.8%)	175,964 (7.7%)
Ethnicity			
Hispanic	522,847 (12%)	243,171 (12%)	279,676 (12%)
Non-Hispanic	3,782,875 (88%)	1,809,761 (88%)	1,973,114 (88%)
Sex			
Female	1,823,077 (41%)	876,077 (41%)	947,000 (41%)
Male	2,647,226 (59%)	1,264,569 (59%)	1,382,657 (59%)
Age group			
<18 Years	484,096 (12%)	231,092 (12%)	253,004 (12%)
18–24 Years	371,632 (9%)	178,436 (9%)	193,196 (9%)
25–34 Years	531,746 (13%)	251,500 (13%)	280,246 (13%)
35–44 Years	430,265 (10%)	200,717 (10%)	229,548 (10%)
45–54 Years	423,323 (10%)	210,098 (10%)	213,225 (9%)
55–64 Years	547,258 (13%)	266,918 (13%)	280,340 (13%)
≥65 Years	1,403,787 (33%)	663,183 (33%)	740,604 (34%)

Trauma outcomes during the study period:

In our multivariable analysis controlling for age, sex, and ethnicity, we observed an overall increase in assaults (Odds Ratio: 1.02, 95% CI: [1.01, 1.03], *p* < 0.001) and firearm-related injuries (1.28 [1.27, 1.29]; p < 0.001) associated with the COVID-19 period (Table 2). In contrast, there was a significant decrease in self-inflicted injuries associated with the COVID period (0.98 [0.97, 0.99]; *p* = 0.039).

**Table 2:** Multivariable logistic regression models predicting injury subtype, all study years combined (2018–2021).

Variable	Assaults			Self-inflicted injuries			Firearm injuries		
	OR	95% CI	*p*	OR	95% CI	*p*	OR	95% CI	*p*
Time period									
Pre-COVID	–	–	–	–	–	–	–	–	–
COVID	1.02	1.01, 1.03	<0.001	0.98	0.97, 1.00	0.039	1.28	1.27, 1.29	<0.001
Race									
AAPI	1.09	1.06, 1.12	<0.001	1.08	1.03, 1.14	0.003	0.80	0.76, 0.84	<0.001
American Indian	3.57	3.47, 3.67	<0.001	1.31	1.23, 1.40	<0.001	1.34	1.27, 1.41	<0.001
Black	5.22	5.18, 5.26	<0.001	0.54	0.53, 0.56	<0.001	6.14	6.07, 6.21	<0.001
Other	1.24	1.23, 1.26	<0.001	0.88	0.85, 0.91	<0.001	1.11	1.09, 1.13	<0.001
White	–	–	–	–	–	–	–	–	–
Ethnicity									
Hispanic	1.69	1.67, 1.71	<0.001	0.73	0.71, 0.75	<0.001	1.44	1.41, 1.46	<0.001
Non-Hispanic	–	–	–	–	–	–	–	–	–
Sex									
Female	0.43	0.43, 0.43	<0.001	0.73	0.72, 0.75	<0.001	0.34	0.34, 0.35	<0.001
Male	–	–	–	–	–	–	–	–	–
Age group									
<18 Years	0.28	0.28, 0.29	<0.001	0.29	0.28, 0.30	<0.001	0.29	0.29, 0.30	<0.001
18–24 Years	–	–	–	–	–	–	–	–	–
25–34 Years	1.07	1.05, 1.08	<0.001	1.06	1.03, 1.08	<0.001	0.69	0.68, 0.70	<0.001
35–44 Years	0.91	0.90, 0.92	<0.001	0.94	0.91, 0.96	<0.001	0.46	0.45, 0.47	<0.001
45–54 Years	0.59	0.58, 0.60	<0.001	0.65	0.63, 0.67	<0.001	0.26	0.25, 0.26	<0.001
55–64 Years	0.32	0.32, 0.32	<0.001	0.39	0.38, 0.41	<0.001	0.12	0.12, 0.13	<0.001
≥65 Years	0.07	0.07, 0.07	<0.001	0.13	0.12, 0.13	<0.001	0.05	0.05, 0.05	<0.001

OR = Odds Ratio; CI = Confidence Interval.

Overall, females experienced lower rates of assault (0.43 [0.43, 0.43]; *p* < 0.001), self-inflicted injuries (0.73 [0.72, 0.75]; *p* < 0.001) and firearm-related injuries (0.34 [0.34, 0.35]; p < 0.001).

AAPI (1.09 [1.06, 1.12]; p < 0.001), American Indians (3.57 [3.47, 3.67]; p < 0.001), Black/African American (5.22 [5.18, 5.26]; *p* < 0.001), and individuals of other races (1.24 [1.23, 1.26]; *p* < 0.001) experienced higher odds of assaults compared to Caucasians. Hispanics showed higher odds of assaults compared to non-Hispanics (1.69 [1.67, 1.71]; p < 0.001) (Table 2).

AAPI (1.08 [1.03, 1.14]; *p* < 0.001) and American Indians (1.31 [1.23, 1.40]; *p* < 0.001) experienced higher odds of self-inflicted injuries compared to Caucasian patients, while lower odds were observed for Black/African American and Other races (Table 2).

American Indians (1.34 [1.27, 1.41]; *p* < 0.001), Black/African American (6.14 [6.07, 6.21]; p < 0.001] and Other races (1.11 (1.09, 1.13); p < 0.001) experienced increased odds of firearm-related injury. Hispanic patients showed higher odds of firearm-related injuries compared to non-Hispanic patients (1.44 [1.41, 1.46]; p < 0.001) (Table 2).

When stratified by time period, racial and ethnic disparities in injury patterns were observed both before and during the COVID-19 pandemic (Table 3). Across assault and firearm-related injuries, odds ratios for several racial and ethnic minority groups were higher during the COVID period compared to the pre-COVID period, demonstrating an overall upward trend over time. These increases were most pronounced for firearm-related injuries among Black/African American patients, whose odds were substantially higher than those of White patients in both periods and increased further during COVID (6.42 [6.33–6.52]; *p* < 0.001).

**Table 3:** Multivariable logistic regression models predicting injury subtype, stratified by time period.

Variable	Assaults						Self-inflicted injuries						Firearm injuries					
	Pre-COVID (2018–2019)			COVID (2020–2021)			Pre-COVID (2018–2019)			COVID (2020–2021)			Pre-COVID (2018–2019)			COVID (2020–2021)		
	OR	95% CI	*p*	OR	95% CI	*p*	OR	95% CI	*p*	OR	95% CI	*p*	OR	95% CI	*p*	OR	95% CI	*p*
Race																		
AAPI	1.13	1.08, 1.17	<0.001	1.06	1.02, 1.10	0.005	1.04	0.96, 1.12	0.3	1.12	1.04, 1.20	0.001	0.78	0.73, 0.84	<0.001	0.81	0.76, 0.86	<0.001
American Indian	3.68	3.54, 3.83	<0.001	3.47	3.35, 3.60	<0.001	1.40	1.28, 1.54	<0.001	1.23	1.12, 1.35	<0.001	1.20	1.10, 1.30	<0.001	1.45	1.36, 1.55	<0.001
Black	5.11	5.05, 5.17	<0.001	5.32	5.26, 5.38	<0.001	0.54	0.52, 0.56	<0.001	0.55	0.53, 0.57	<0.001	5.79	5.70, 5.89	<0.001	6.42	6.33, 6.52	<0.001
Other	1.26	1.24, 1.29	<0.001	1.23	1.20, 1.25	<0.001	0.87	0.82, 0.91	<0.001	0.88	0.84, 0.93	<0.001	1.11	1.07, 1.14	<0.001	1.11	1.08, 1.15	<0.001
White	–	–	–	–	–	–	–	–	–	–	–	–	–	–	–	–	–	–
Ethnicity																		
Hispanic	1.68	1.65, 1.71	<0.001	1.71	1.68, 1.74	<0.001	0.72	0.69, 0.75	<0.001	0.73	0.70, 0.76	<0.001	1.41	1.37, 1.45	<0.001	1.46	1.43, 1.50	<0.001
Non-Hispanic	–	–	–	–	–	–	–	–	–	–	–	–	–	–	–	–	–	–
Sex																		
Female	0.42,	0.41, 0.42	<0.001	0.44	0.43, 0.44	<0.001	0.72	0.70, 0.74	<0.001	0.74	0.72, 0.77	<0.001	0.33	0.32, 0.33	<0.001	0.35	0.34, 0.36	<0.001
Male	–	–	–	–	–	–	–	–	–	–	–	–	–	–	–	–	–	–
Age group																		
<18 Years	0.26	0.26, 0.27	<0.001	0.30	0.29, 0.31	<0.001	0.27	0.25, 0.29	<0.001	0.30	0.29, 0.32	<0.001	0.27	0.26, 0.28	<0.001	0.31	0.30, 0.31	<0.001
18–24 Years	–	–	–	–	–	–	–	–	–	–	–	–	–	–	–	–	–	–
25–34 Years	1.06	1.04, 1.08	<0.001	1.07	1.06, 1.09	<0.001	1.08	1.04, 1.12	<0.001	1.03	0.99, 1.07	0.10	0.69	0.68, 0.71	<0.001	0.68	0.67, 0.69	<0.001
35–44 Years	0.89	0.88, 0.91	<0.001	0.93	0.92, 0.95	<0.001	0.98	0.94, 1.02	0.3	0.90	0.86, 0.94	<0.001	0.46	0.45, 0.47	<0.001	0.46	0.45, 0.47	<0.001
45–54 Years	0.57	0.56, 0.58	<0.001	0.61	0.60, 0.62	<0.001	0.68	0.65, 0.71	<0.001	0.62	0.59, 0.65	<0.001	0.26	0.25, 0.27	<0.001	0.25	0.25, 0.26	<0.001
55–64 Years	0.31	0.30, 0.32	<0.001	0.33	0.32, 0.34	<0.001	0.43	0.41, 0.45	<0.001	0.36	0.35, 0.38	<0.001	0.13	0.13, 0.14	<0.001	0.12	0.11, 0.12	<0.001
≥65 Years	0.06	0.06, 0.06	<0.001	0.07	0.07, 0.07	<0.001	0.14	0.13, 0.14	<0.001	0.12	0.11, 0.13	<0.001	0.06	0.05, 0.06	<0.001	0.04	0.04, 0.05	<0.001

## Discussion

The COVID-19 pandemic led to significant social unrest, driven by economic instability, social isolation, and heightened community tensions [[16], [17], [18]]. These stresses were exacerbated by strained healthcare systems, disparities in access to resources, and increased public awareness of social and racial inequalities, contributing to widespread protests and conflicts during this period [[19], [20], [21], [22], [23], [24]]. The impact of this global health crisis and social unrest on society was manifested by the increases in assaults, gun violence, and incidence of self harm. These effects are often magnified in racial and ethnic minority groups, who often experience these disparities in our healthcare system [25]. It is imperative to study the effects of the pandemic on violent trauma on a societal level with a special focus on the most vulnerable communities to guide future policies.

An increase in assaults, including firearm related injuries, was reported in multicenter and single trauma center-based studies following the COVID-19 pandemic [6,[26], [27], [28], [29]]. Our NTDB study supports these findings with a 0.5% increase in both assaults and firearm-related injuries following the beginning of the pandemic. The effect on specific racial populations was studied by Strassle et al. (2021), identifying a substantial increase in assault hospitalizations at the start of the pandemic, particularly among Black/African American populations and males aged 18–44 [2]. Additionally, in our recent assessment of pre-hospital assault data using national emergency medical services, we found increases in assault rates during the COVID period, specifically among AAPI and Hispanic patients [30]. Similarly, our study identified an increase in the incidence of assaults with higher odds ratios among the AAPI, American Indian, Black, and Hispanic communities compared to the Caucasian/non-Hispanic populations. The highest odds ratios were seen in the Black and American Indian communities. This disparity in assault patterns is likely multifactorial in origin, which included societal tensions like racism as well as economic stressors like job losses. However, our assault data shows that the minority populations of America felt the predominant brunt of the social unrest of 2020. More studies are needed to elaborate on specific causes of this to prevent this in the future.

While there was an overall increase in assaults during the COVID-19 pandemic, minority communities, such as the AAPI community, experienced more of an impact with increased rates compared to Caucasian communities. These trends in the AAPI community were noted to persist from 2020 into 2021, correlating with increased hate crimes toward this group reported by police during the pandemic period. [30,31] These initial trends similarly observed in our study could be explained by negative attitudes toward the AAPI communities during the COVID-19 pandemic [32]. However, it remains a challenge to determine the proportion of hate crimes among the assaults given that TQIP data does not include the intentions behind the assaults.

In stratified models, these racial and ethnic disparities in assaults and firearm-related injuries were already substantial in the pre-COVID period and were generally similar or modestly larger during the COVID period, particularly for Black/African American and American Indian patients. In contrast, the odds of self-inflicted injuries among AAPI patients became significantly higher than in White patients during the COVID period, suggesting a pandemic-related increase in self-inflicted harm in this community. Taken together, these findings indicate that the COVID-19 pandemic largely intensified these disparities rather than creating them.

There was a notable increase in firearm-related injuries during the COVID-19 pandemic, with emergency room visits and hospitalizations rising significantly [[33], [34], [35], [36]]. Studies have shown that the risk for these firearm-related injuries was particularly high among young men, especially Black men [34]. Other research indicated that Indigenous and Hispanic individuals, along with non-Hispanic, Black/African American males, experienced higher rates of firearm-related injuries compared to Caucasians [37,38]. Our findings are consistent with current literature, identifying 6.14 OR of firearm related injury in Black/African American and 1.34 OR in American Indian communities during the pandemic compared to their Caucasian counterparts. Our findings also supported a statistically significant increase in gun violence in the ethnically Hispanic population. The increased rate of firearm injuries is complex in etiology but also coincided with a significant increase in firearm purchases during the pandemic causing a significant rise in exposure of firearms at home during a particularly disruptive time of heightened stress and social instability [35].Our data shows that male Blacks, American Indians and Hispanics were disproportionately affected by these changes.

Disparities among trauma care and outcomes, such as mortality rates and discharge dispositions have been found among different races/ethnicities. Firearm violence in the US disproportionately affects different populations. These differences may stem from inequalities and inequities from long standing systemic racism. Firearm homicides were found to be the leading cause of death of young Black males between the ages of 15 and 34 years old [39]. Knopov et al. (2019) studied disparities of firearm homicides in regards to racial residential segregation. They found that states with more segregated Black and Caucasian populations had markedly larger gaps in firearm homicide rates, with higher rates among Blacks compared to Caucasians in these segregated communities [40]. This study adds to the literature regarding disparities in violent assaults and crimes among Black and Caucasian communities. [[41], [42], [43]]

Poverty, unemployment, and income inequality are consistently linked with higher rates of firearm violence. Communities facing economic strain (e.g. unemployment, high poverty, housing instability) have elevated risks for homicide and suicide from firearms. These findings were more pronounced among Black/African Americans and younger males. [[44], [45], [46]] However, while economic hardship contributes significantly to firearm violence, the opposite is also true: measures of economic well-being can offer protective effects. Higher social capital and greater upward economic mobility are associated with lower firearm homicide rates [45]. These findings point to economic deprivation as a cause of violence. Further studies are needed to assess social, economic and cultural risk factors that can be mitigated to protect these at risk communities.

As with firearm-related injuries, disparities in self-inflicted injuries are also influenced by social determinants of health. Individuals with lower socioeconomic status face a higher risk of suicide by firearms, while generational upward mobility has been shown to reduce this risk [47]. States with higher relative minimum wages experienced lower suicide rates [48]. Income disparities– with racial and ethnic minority groups generally earning less than Caucasians– may be a factor contributing to the differences in self-inflicted injuries observed across racial and ethnic groups. These socioeconomic disparities were further amplified during the COVID-19 pandemic, when minority communities experienced higher unemployment rates and received a smaller share of COVID stimulus funds compared to Caucasians [20,[49], [50], [51], [52]]. These socioeconomic differences worsened by the COVID-19 pandemic may help explain the noticeable rise in self-inflicted injuries among minority groups in our study, further suggesting that economic disadvantage serves as a structural driver of self-harm. Policies aimed at improving economic opportunities, alongside implementing targeted suicide prevention programs could help reduce these disparties.

The COVID-19 pandemic has profoundly impacted mental health across the nation [53]. Recent studies have highlighted increasing trends in emergency department visits, repeated injuries among youth, increased risk of self-inflicted injuries among women, and links between major psychiatric illnesses and self-inflicted gun violence injuries [[54], [55], [56], [57], [58]]. Multiple studies reported a notable rise in self-inflicted injuries, as seen by the increase in emergency department visits for mental health conditions, suicide attempts, overdoses, and violence, demonstrating a shift in healthcare needs following the COVID-19 pandemic [[59], [60], [61]]. Additionally, studies have also demonstrated an increase in admission for trauma interventions after suicide attempts and self-inflicted burn injuries [61,62]. Conversely, Seng et al. (2023), utilizing data from a single state trauma registry, had similar findings with no significant increase in self-inflicted injuries overall during the pandemic [63]. In another study, Moore A. et al. analyzing the effect of the pandemic on assaults found a decrease in self-inflicted injuries [64]. Our analysis of a national data set revealed a stable rate of self-inflicted injuries at 1.3% of the entire trauma population. Trauma registry based studies only capture patients evaluated and treated for traumatic injuries at trauma centers as a result of self-harm and thus likely do not reflect all instances of self-harm (ie pill ingestion, minor trauma seen at urgent care, successful suicide, etc). Given the increased mental health challenges seen because of the COVID-19 pandemic, our findings may not fully reflect the impact of COVID-19 pandemic on mental health and self-harm [65,66].

Our findings on minority populations in the context of self-inflicted injuries during the pandemic add to the growing body of literature [67]. Despite the similar incidence in self-inflicted injuries before and during the COVID period, once disaggregated by race, our analysis revealed significant differences particularly among Asian American Pacific Islanders. After controlling for age, sex, and COVID years, AAPIs and American Indians were found to have higher odds of self-inflicted injuries compared to Caucasian patients. During COVID-19, we saw an increase in discrimination against AAPI which may have led to an increased suicidality in this group [68]. Furthermore, a study published by Nie et al. (2024) found that internalized racism had a significant effect on suicidal ideation among Asian and Asian American adults in the US, even surpassing the influence of external racism and other mental health factors such as anxiety and loneliness [69]. These findings on how internalized racism among AAPIs can contribute to mental health challenges correlates with the increase in self-inflicted injuries we see among AAPIs during the pandemic in our study population. Healthcare providers and community leaders should consider these cultural specific factors in the future care of minority populations. The disproportionate increase in mental health emergencies and suicidal behaviors highlights the critical need for nuanced and targeted support and interventions for all populations, specifically those most vulnerable [70].

Our study shares valuable knowledge on racial/ethnicity disparities among patients with firearm-related injuries and self-inflicted injuries; however, it is not without limitations. One limitation is that our study utilizes TQIP, a national dataset, which does not disaggregate into urban, suburban and rural settings of the incidents. Additionally, the dataset lacks information on the geographical location where the traumas occurred. Such data could provide valuable insights into injury incidence patterns and help inform firearm policies, as well as guide the allocation of trauma prevention resources for policymakers. TQIP data also does not disaggregate the AAPI community into its various subgroups (i.e. East Asian, South Asian, Pacific Islander, etc.), which is known to have different health trends and outcomes among its subgroups [71,72]. Additionally with different subgroups of AAPI reporting varying experiences with COVID-19-related discrimination, disaggregated data to better evaluate trauma trends would help more effectively direct resources to the communities most affected. [73] Also, while the TQIP database is robust, we cannot assign intent of the data. So while we can comment on the victims, we cannot fully comment on how or why the injury took place. Another limitation is that we do not have the medical and socioeconomic history of patients limiting the scope of analyzing causal factors of injury. Furthermore, our access to TQIP was limited to the defined study period, so we were unable to evaluate longer-term pre-COVID secular trends in assaults and firearm-related injuries. As a result, we cannot determine whether the increases observed during the pandemic represent a true inflection point or an acceleration of pre-existing trends. Lastly, TQIP data does not capture traumatic injuries treated at non-trauma centers or outpatient facilities. It also does not encompass patients that did not present to any healthcare facilities for treatment of injuries sustained from assaults or self-harm. Future studies are needed to address these limitations to provide a more comprehensive understanding of the factors influencing firearm-related and self-inflicted injuries.

## Conclusion

During the COVID-19 pandemic, we saw a marked increase in assaults and firearm-related injuries, alongside a steady rate of decrease in self-inflicted injuries. Racial minorities had significantly higher odds of assaults, with Black/African American patients having particularly higher odds of assaults and firearm-related injuries. In contrast to other racial minorities and to the general population, Asian American Pacific Islanders experienced higher rates of self-inflicted injuries during the pandemic. These findings highlight the critical need for further research to understand the factors contributing to health disparities faced by minority racial and ethnic groups. Moreover, our results emphasize the need for more comprehensive programs aimed at preventing violence and addressing the unique needs of ethnic and racial minority communities.

## Notes

This study was originally presented at the 9th Annual Society of Asian Academic Surgeons meeting on September 5, 2024 in New Orleans, LA.

## CRediT authorship contribution statement

**Veronica Layrisse Landaeta:** Visualization, Validation, Methodology, Investigation, Data curation, Conceptualization, Writing – review & editing, Writing – original draft. **Shahenda Khedr:** Visualization, Validation, Project administration, Methodology, Investigation, Conceptualization, Writing – review & editing, Writing – original draft. **Victoria Yuan:** Visualization, Investigation, Writing – review & editing, Writing – original draft. **Eshani Pareek:** Validation, Methodology, Investigation, Formal analysis, Data curation. **Debra D'Angelo:** Validation, Methodology, Investigation, Formal analysis, Data curation. **Elizabeth Zhao:** Visualization, Investigation, Writing – review & editing, Writing – original draft. **Gala Cygiel:** Visualization, Investigation, Writing – review & editing, Writing – original draft. **Konstantin Khariton:** Visualization, Supervision, Investigation, Writing – review & editing. **Steven Y. Chao:** Visualization, Supervision, Investigation, Writing – review & editing.

## Ethics approval

Our study evaluates firearm and self-inflicted injury risks across different races and ethnicities. The study used publicly available, de-identified data and did not involve human or animal subjects. Therefore, ethics committee approval and informed consent were not required.

## Funding sources

This research did not receive any specific grant from funding agencies in the public, commercial, or not-for-profit sectors.

## Declaration of competing interest

The authors declare that they have no known competing financial interests or personal relationships that could have appeared to influence the work reported in this paper.
